# Involvement of Gastrin-Releasing Peptide Receptor in the Regulation of Adipocyte Differentiation in 3T3-L1 Cells

**DOI:** 10.3390/ijms19123971

**Published:** 2018-12-10

**Authors:** Mi-Kyoung Kim, Hyun-Joo Park, Yeon Kim, Soo-Kyung Bae, Hyung Joon Kim, Moon-Kyoung Bae

**Affiliations:** 1 Department of Oral Physiology, BK21 PLUS Project, School of Dentistry, Pusan National University, Yangsan 50610, Korea; eenga@naver.com (M.-K.K.); phj3421@hanmail.net (H.-J.P.); graceyeon88@gmail.com (Y.K.); hjoonkim@pusan.ac.kr (H.J.K.); 2 Department of Dental Pharmacology, BK21 PLUS Project, School of Dentistry, Pusan National University, Yangsan 50610, Korea; skbae@pusan.ac.kr

**Keywords:** gastrin-releasing peptide, gastrin-releasing peptide receptor, adipocyte differentiation

## Abstract

Gastrin-releasing peptide (GRP), a member of bombesin-like peptides, and its receptor (GRP-R) play an important role in various physiological and pathological conditions. In this work, we investigated the role of GRP-R on adipogenesis in 3T3-L1 adipocytes. The expression of GRP-R was significantly increased during the adipocyte differentiation of 3T3-L1 cells. The inhibition of GRP-R by the antagonist RC-3095 affected adipogenesis in 3T3-L1 cells, which reduced lipid accumulation and regulated the expression of adipogenic genes. Moreover, cyclic AMP response element-binding protein (CREB) directly bound to the GRP-R promoter upon exposure to adipogenic stimuli. The down-regulation of GRP-R by the knockdown of CREB inhibited adipocyte differentiation of 3T3-L1 cells. Together these results suggest that the regulation of GRP-R activity or expression has an influence on adipogenesis through regulating adipogenic related genes.

## 1. Introduction

The family of mammalian bombesin receptors is composed of three receptors: Gastrin-releasing peptide receptor (GRP-R/BB2), neuromedin B receptor (NMB-R/BB1), and the orphan bombesin receptor subtype-3 (BRS-3/BB-3) [1]. These mammalian bombesin receptors are specific G-protein-coupled receptors that mediate the diverse actions of bombesin-like peptides (BLPs) in autocrine, paracrine, or neuroendocrine systems [2]. BLPs, including gastrin-releasing peptide (GRP) and neuromedin B (NMB), have been identified in mammalian tissues. The interaction of GRP-R with GRP influences a wide range of physiological processes including thermoregulation, gastric and pancreatic secretion, stress responses, and food intake and satiety [3]. Increasing evidence has suggested that GRP and its receptor act as a mitogen, morphogen, and proangiogenic factor in many types of cancer, and an inflammatory mediator in inflammatory cells and vascular cells [4,5].

GRP is widely expressed and secreted in the neurons of the brain, spinal cord, and gastrointestinal tract as well as in some tumors including small cell lung cancer (SCLC), prostate cancer, and esophageal squamous cell carcinoma [6,7]. However, the expression of GRP-R in normal tissues has not been fully determined yet; GRP-R has been reported to be expressed in some central and peripheral tissues including the brain, stomach, pancreas, and adrenal cortex [8]. Meanwhile, NMB-R is detected in visceral adipocytes, and NMB is also expressed in adipose tissue, which is involved in the regulation of energy balance [9,10]. However, the expression and role of GRP and GRP-R have not previously been reported in adipose tissues.

In our present study, we investigated the expression of GRP-R during adipogenesis and the underlying molecular mechanisms, and characterized the action of the GRP-R antagonist on the adipogenic differentiation of 3T3-L1 preadipocytes.

## 2. Results

### 2.1. GRP-R Expression Is Increased during Adipocyte Differentiation

**Figure 1:**
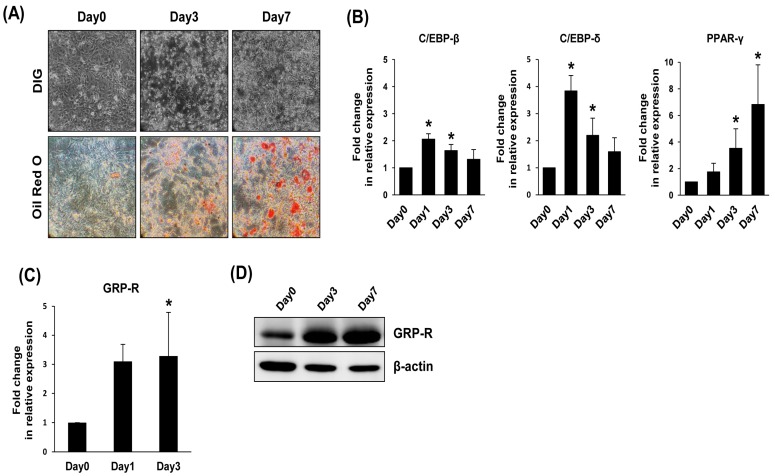
The expression of GRP-R (Gastrin-releasing peptide receptor) in adipocyte differentiation of 3T3-L1 cells. Preadipocyte 3T3-L1 cells were induced to differentiate by the addition of DMI (DEX, IBMX, and insulin) for the indicated times. (**A**) During adipogenic differentiation, changes in cell morphology were observed by a phase contrast microscope at 200× magnification, and oil red O staining was performed. (**B**) Total RNA was isolated during adipogenesis. The adipocyte specific markers (C/EBP-β, C/EBP-δ, and PPAR-γ) were investigated by real-time PCR. The level of expression was normalized to 36B4. Each value represents the mean ± SD from three independent experiments. * *p* < 0.05 compared to Day 0. (**C**) The mRNA expression of GRP-R during 3T3-L1 differentiation was measured by real-time PCR. * *p* < 0.05 compared to Day 0. (**D**) The level of GRP-R protein was detected using the specific antibody. β-actin was used as the internal control.

To determine the expression pattern of GRP-R during adipocyte differentiation, we prepared preadipocyte 3T3-L1 and differentiating/differentiated 3T3-L1 cells. For adipocyte differentiation, 3T3-L1 preadipocytes were stimulated with differentiation medium for 7 days. As shown in Figure 1A, the cell morphology changed to adipocyte-like cells, and lipid droplets were accumulated. Consistent with the observation of lipid droplet staining with Oil Red O, the mRNA expression of the adipogenic marker genes, CCAAT/enhancer binding protein-β (C/EBP-β), CCAAT/enhancer binding protein-δ (C/EBP-δ), and peroxisome proliferator-activated receptor-γ (PPAR-γ), was significantly increased upon exposure to adipogenic stimuli (Figure 1B). During differentiation, the cooperative action of C/EBP-β, C/EBP-δ, and PPAR-γ stimulates adipogenesis [11]. Interestingly, the mRNA expression of GRP-R was significantly induced with adipocyte differentiation (Figure 1C). Similarly, the protein level of GRP-R was dramatically increased during adipogenic differentiation (Figure 1D). 

### 2.2. GRP-R Antagonist Modulates Adipocyte Differentiation

**Figure 2:**
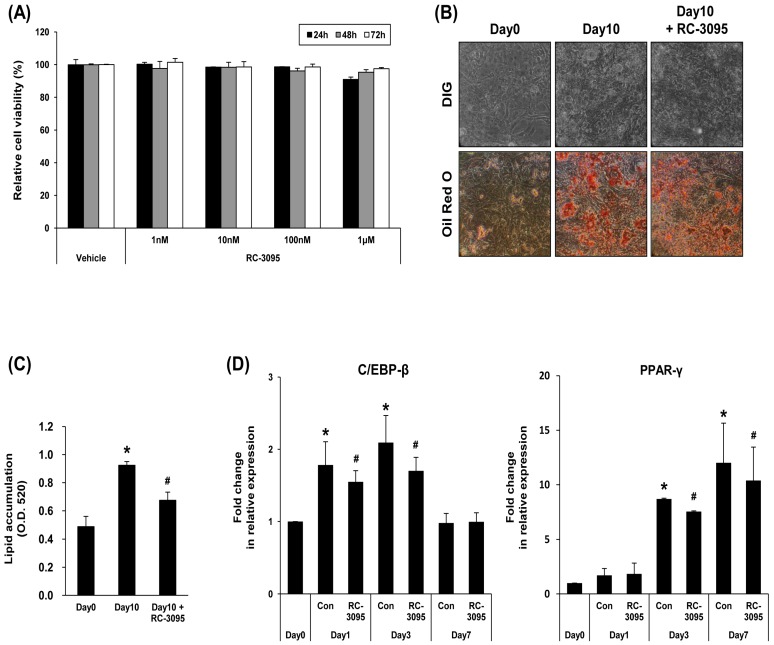
RC-3095 modulates the adipocyte differentiation of 3T3-L1 cells. (**A**) 3T3-L1 cells were incubated with various concentrations of RC-3095 for 24, 48, or 72 h, respectively. Cell viability was determined by MTT (3-[4-dimethylthiazol-2-yl]-2,5-diphenyltetrazolium bromide) assay. Dimethyl sulfoxide (DMSO) was used as a solvent for RC-3095. (**B**) During differentiation, RC-3095 (1 µM) was co-treated with DMI. Adipogenic cells were observed and stained with Oil Red O under a phase-contrast microscope at 200× magnification. (**C**) Oil Red O was extracted and quantitatively analyzed. Each value represents the mean of at least three independent experiments. * *p* < 0.01 was compared to Day 0. ^#^ *p* < 0.05 was compared to the positive control. (**D**) The effect of RC-3095 on adipogenic differentiation markers was analyzed by real-time PCR using primers specific to C/EBP-β or PPAR-γ. * *p* < 0.01 was compared to Day 0. ^#^ *p* < 0.05 was compared to the positive control.

To investigate the role of GRP-R in adipocyte differentiation, 3T3-L1 preadipocytes were cultured in the presence of RC-3095, a specific antagonist of GRP-R [12]. Various doses of RC-3095 (1 nM, 10 nM, 100 nM, or 1 μM) did not affect the viability of 3T3-L1 cells (Figure 2A). In this study, 3T3-L1 cells under differentiation were incubated with 1 μM of RC-3095. The effect of RC-3095 on 3T3-L1 preadipocyte differentiation was observed by cell morphology changes and Oil Red O staining (Figure 2B). RC-3095 reduced lipid accumulation on day 10 of 3T3-L1 preadipocyte differentiation (Figure 2C). Furthermore, we confirmed the effect of RC-3095 on the expression of adipogenic marker genes. As shown in Figure 2D, the expressions of C/EBP-β or PPAR-γ were mildly reduced by RC-3095 in separate repeated experiments. In addition, the treatment of 3T3-L1 cells with GRP slightly increased the accumulation of lipid droplets and the expressions of adipogenic markers during adipocyte differentiation (Figure S1).

### 2.3. Downregulation of GRP-R by CREB Knockdown Inhibits the Adipocyte Differentiation of 3T3-L1 Cells

**Figure 3:**
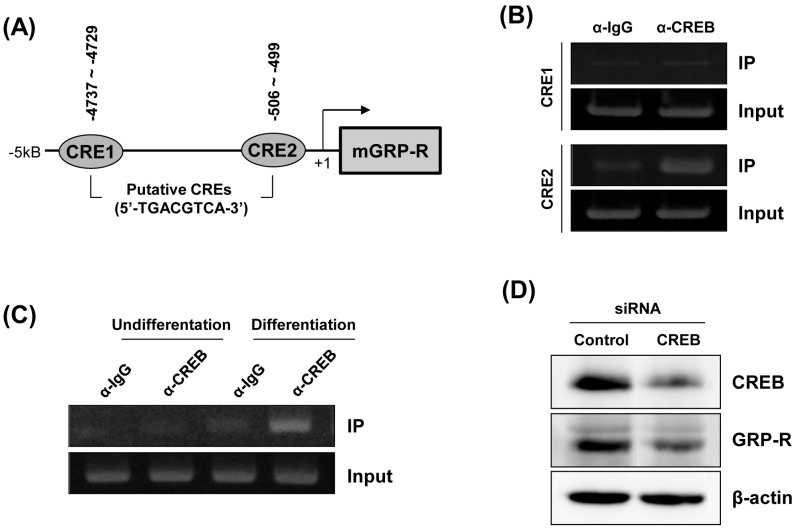

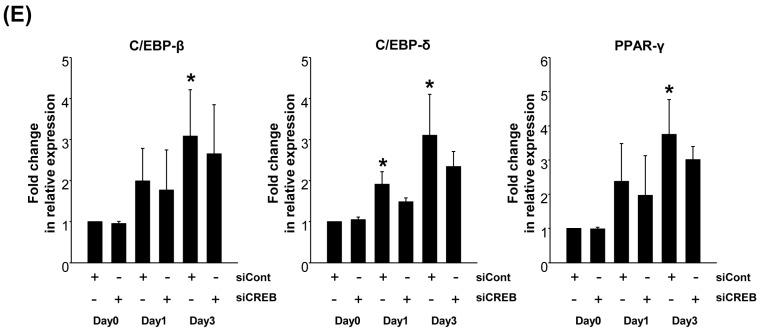
Involvement of CREB-dependent GRP-R in adipogenesis. (**A**) Localization of putative CREs within the ~5 kb fragment of the 5′–flanking region of the mouse GRP-R gene. Putative CRE sites were defined by the core sequence 5′-TGACGTCA-3′. (**B**) The sonicated cell lysate from differentiated 3T3-L1 cells was immunoprecipitated with antibodies against control IgG or CREB. A sonicated cell lysate was used as an input control. The two putative CRE binding sites (CRE1, −4737–−4729 and CRE2, −506–−499) were amplified by PCR using specific primers to the CRE1 or CRE2 sequence. (**C**) The sonicated cell lysate from undifferentiated or differentiated 3T3-L1 cells were cultured as described and subjected to ChIP assay. IgG or CREB antibody was used to precipitate sonicated chromatin. (**D**) 3T3-L1 cells were transfected with negative control siRNA or CREB siRNA for 72 h. The expression of CREB or GRP-R protein was confirmed by Western blot assay using CREB or GRP-R antibodies, respectively. (**E**) Control siRNA or CREB siRNA-transfected 3T3-L1 cells were incubated with DMI for indicated days. Total RNA was purified and analyzed by real-time PCR using specific primers for C/EBP-β, C/EBP-δ, or PPAR-γ. * *p* < 0.05 compared to Day 0.

To further confirm the role of GRP-R in adipocyte differentiation, we tried to design and introduce different GRP-R siRNA target sequences into 3T3-L1 cells, but could not obtain efficient GRP-R siRNA constructs to knockdown the mouse GRP-R gene. It has been reported that cyclic AMP response element (CRE) sites exist within the promoter of GRP-R and that cyclic AMP response element binding protein (CREB) is critical for the transcriptional regulation of GRP-R expression [13,14]. To evaluate the binding of CREB to the GRP-R promoter, chromatin immunoprecipitation (ChIP) was performed on differentiated 3T3-L1 adipocytes. First, using an extensive analysis of the 5′–flanking region of the mouse GRP-R gene, we identified two putative CRE sites, CRE1 (−4737–−4729) and CRE2 (−506–−499) within 5 kb of the 5′–flanking promoter region of the mouse GRP-R gene (Figure 3A). The recruitment of CREB to the promoter regions was assessed by PCR using two primer sets encompassing CRE1 and CRE2 sequences, respectively. Of the two CRE sites, CREB specifically binds with high affinity to the CRE2 (−506–−499) region within the mouse GRP-R promoter (Figure 3B). In addition, to investigate whether adipogenic differentiation stimuli affect the interaction between CREB and the GRP-R promoter, we performed a ChIP assay with 3T3-L1 cell lysates before and after the induction of differentiation. As shown in Figure 3C, PCR products corresponding to the interaction of CREB with the CRE2 region of the GRP-R promoter were only detected in differentiated 3T3-L1 adipocytes. This result indicated that the recruitment of CREB to the CRE2 region within the GRP-R promoter was essential for the induction of the GRP-R gene during the differentiation of 3T3-L1 cells. Next, to demonstrate whether GRP-R expression is modulated by CREB, 3T3-L1 cells were transfected with CREB-targeting siRNA. The silencing effect of the CREB siRNA transfection was confirmed by a decrease in the protein expression level of CREB. As shown in Figure 3D, the knockdown of CREB suppressed the protein expression of GRP-R. Next, we elucidated whether the downregulation of CREB decreases the adipogenesis of 3T3-L1 cells. In the presence of CREB siRNA, the 3T3-L1 preadipocytes were differentiated into adipocytes in adipogenic differentiation medium. We then performed real-time quantitative PCR for expression analysis of adipogenic-related factors. As shown in Figure 3E, the induction of adipogenic markers, C/EBP-β, C/EBP-δ or PPAR-γ, were downregulated by the transfection of CREB siRNA.

### 2.4. Expression of GRP-R and GRP in Adipose Tissues of High-Fat Diet-Induced Obese Rats

**Figure 4:**
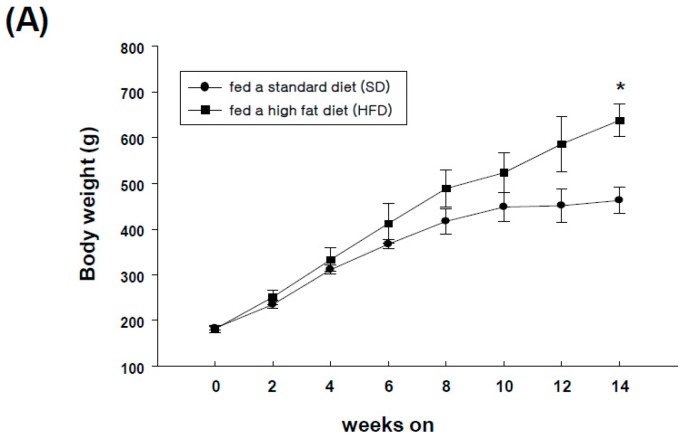

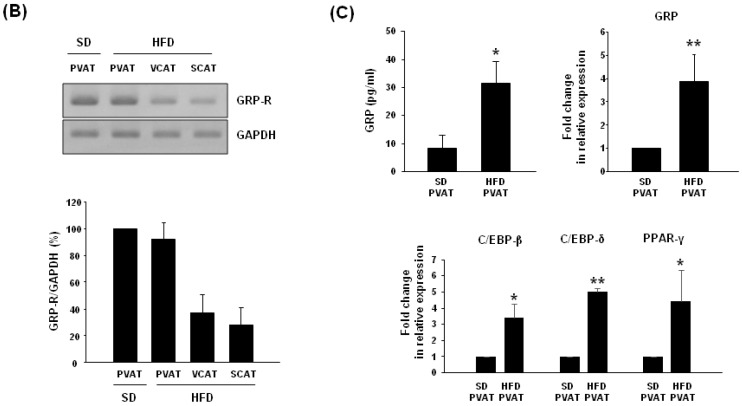
Expression of GRP-R and GRP in adipose tissues from obese rats. (**A**) The SD (standard diet) group was fed a standard diet, and the HFD (high-fat diet) group was fed a high-fat diet for 14 weeks. The body weight was measured once every two weeks. (**B**) All rats were sacrificed at week 14, and subcutaneous (SCAT), visceral (VCAT) or perivascular adipose tissues (PVAT) from the HFD group and perivascular adipose tissues from SD group were obtained. The total RNA was extracted from each adipose tissue and then analyzed by RT-PCR using primers specific for GRP-R. GAPDH served as an internal control (top). The graph shows the densitometric analysis of the relative GRP-R mRNA levels. The GRP-R expression from the PVAT of SD rats was set to 100% (bottom). (**C**) Conditioned medium from the PVAT of HFD-induced obese rats or from the PVAT of SD rats was prepared, respectively, and then, the secreted GRP protein in rat perivascular adipose tissue-conditioned medium was quantified by ELISA. The mRNA levels of GRP, C/EBP-β, C/EBP-δ, and PPAR-γ in PVAT of HFD-induced obese rats or in PVAT of SD rats were quantitatively analyzed by real-time PCR using specific primers for GRP, C/EBP-β, C/EBP-δ, or PPAR-γ. * *p* < 0.05, ** *p* < 0.01 was compared to the PVAT of SD rats.

To determine the expression of GRP-R in adipose tissues *in vivo*, we developed a high-fat diet (HFD)-induced obese rat model for 14 weeks and monitored body weight. All rats became obese with a body weight averaging 1.5–fold higher than rats fed a standard diet (SD) (Figure 4A). We next examined the levels of expression of GRP-R in the adipose tissues of HFD-induced obese rats. GRP-R mRNA was present in subcutaneous, visceral, and perivascular adipose tissues. The GRP-R mRNA level was higher in perivascular adipose tissues than in visceral or subcutaneous adipose tissues. Meanwhile, the mRNA expression level of GRP-R in perivascular adipose tissues was similar between SD and HFD-induced obese rats (Figure 4B). However, the secretion of GRP protein was significantly increased in conditioned medium from the perivascular adipose tissue (PVAT) of HFD-induced obese rats compared to SD rats. The mRNA levels of GRP and adipogenic markers were also consistently upregulated in the PVAT of HFD-induced obese rats compared to those in standard diet rats (Figure 4C). These results suggest that GRP can be actively released from the PVAT of obese rats.

## 3. Discussion

The promoter region of the human and mouse *GRP-R* gene has been determined in human gastrointestinal cancer cell lines such as HuTu-80 and Caco-2, prostatic epithelial cancer cells, and Swiss 3T3 cells [13,15]. Xiao et al. identified CRE, AP-1, and Oct-1 sites within the 5ʹ–flanking region of the human GRP-R gene [16]. The transcriptional regulation of the human *GRP-R* gene requires the binding of the CREB transcription factor to two distinct CRE sites at 112 bp and 1108 bp upstream of the transcription start site [13]. The mouse *GRP*-*R* gene was also identified, which includes all three exons, parts of the introns, and part of the 5ʹ- and 3ʹ-flanking regions as in the human gene [14]. Two kb of the 5′-flanking region of the mouse *GRP*-*R* gene (between positions −2569 and +1) was analyzed and revealed the presence of a cis-regulatory consensus sequence, including TATA-like motifs, a SP-1 site, AP-1 and AP-2 sites, and one CRE site [14]. In the present study, two putative CRE sites, between −4737 to −4729 bp and −506 to −499 bp, have been identified by computer-based analysis of the 5ʹ-flanking region of the mouse *GRP-R* gene (between positions −5426 and +1). Further, we demonstrated the interaction between the CREB protein and CRE (−506–−499) region of the mouse *GRP*-*R* promoter in differentiated 3T3-L1 adipocytes. Several lines of evidence have indicated that CREB plays an essential role as a transcriptional activator in the different stages of the differentiation of preadipocytes to adipocytes [17,18]. Further investigation will be needed to determine whether the transcriptional activity of the mouse *GRP*-*R* promoter can be regulated during adipocyte differentiation and the binding of functional CREB to CRE site(s) is required for the transcriptional activation of the *GRP-R* promoter in differentiating 3T3-L1 adipocytes.

Adipose tissue functions not only as energy storage but also an endocrine organ that secretes a variety of bioactive substances such as adipokines [19]. Obesity is defined as an accumulation of excessive adipose tissue that produces dysregulated adipokines, which contributes to the pathogenesis of obesity-related diseases, including type 2 diabetes and cardiovascular disease [20,21]. Most blood vessels are surrounded by perivascular adipose tissue (PVAT), which was considered a passive structural support for the vasculature [22]. More recently, it has been reported that PVAT actively regulates vascular tone and function, and produces various adipokines with a paracrine vascular effect due to the proximity of PVAT to the vascular wall [23]. Obesity causes the excessive accumulation of inflamed and dysfunctional PVAT, which is regarded as a major risk factor for the development of cardiovascular diseases [24,25]. We report in this study that secreted GRP levels were elevated in PVAT supernatant in rats fed a high-fat diet, compared to PVAT in rats fed a normal diet. We previously reported that GRP induces endothelial dysfunction and the proliferation/migration of vascular smooth muscle cells, the main events of the progression of atherosclerosis [26,27]. Therefore, we speculate that obese PVAT-derived GRP acts as a pro-inflammatory adipokine to stimulate vascular inflammatory responses in the adjacent vascular wall (vascular endothelial and vascular smooth muscle cells). We did not detect a high level of GRP in differentiated 3T3-L1 adipocytes (Figure 1A). The profiles of the secretome from perivascular fat cells are different than those from subcutaneous and visceral fat cells [28]. PVAT is comprised of many cell types, not only adipocytes but also a stromal vascular fraction that includes mesenchymal stem cells, vascular endothelial cells, macrophages, and leukocytes [29]. Therefore, the expression pattern of GRP needs to be determined in order to understand its role in perivascular adipocytes in comparison with visceral or subcutaneous adipocytes and characterize which type of cells in obese PVAT predominantly produce and secrete GRP. In conclusion, these results indicate that the upregulation of GRP-R during adipogenesis is mediated by activated CREB. We also demonstrated that a GRP-R antagonist modulates the differentiation of 3T3-L1 cells into mature adipocytes. Further in vivo studies will be necessary to confirm the critical role of GRP or GRP-R in the molecular regulation of adipose tissue function in normal and pathological states.

## 4. Materials and Methods

### 4.1. Reagents

RC-3095 and GRP were provided by Sigma-Aldrich (St. Louis, MO, USA). Dulbecco’s modified Eagle’s medium (DMEM), fetal bovine serum (FBS), and calf serum (CS) were obtained from Gibco Life Technologies (Grand Island, NY, USA). 3-isobutyl-1-methylxanthine (IBMX), dexamethasone, insulin, and Oil Red O solution were purchased from Sigma-Aldrich (St. Louis, MO, USA). 3-[4-Dimethylthiazol-2-yl]-2,5-diphenyltetrazolium bromide (MTT) was obtained from Sigma-Aldrich. Rabbit polyclonal GRP-R and rabbit monoclonal CREB antibodies were obtained from Santa Cruz Biotechnology (Dallas, TX, USA) and Cell Signaling Technology (Danvers, MA, USA), respectively. β-actin antibody was purchased from Bioworld Technology (St. Louis Park, MN, USA).

### 4.2. Cell Culture

Mouse 3T3-L1 preadipocytes were grown in DMEM containing 10% CS at 37 °C in a humidified 5% CO_2_ incubator. For adipocyte differentiation, 3T3-L1 cells were maintained 2 days post-confluence. Cell differentiation was then induced with DMI (1 μM dexamethasone, 0.5 mM IBMX, and 5 μg/mL insulin) in DMEM with 10% FBS for 72 h. Afterward, the cells were maintained in DMEM supplemented with 10% FBS and 5 μg/mL insulin for 4 days. This medium was changed every 2 days. RC-3095 was added with every medium change during the differentiation period.

### 4.3. Oil Red O Staining

At the end of differentiation, the cells were washed with phosphate-buffered saline (PBS) and fixed with 10% formalin for 1 h. The cells were rinsed with 60% isopropanol and completely dried. The lipid droplets were stained with Oil Red O solution for 10 min. After staining, the Oil Red O solution was removed, and the plates were washed with water and dried. Stained lipid droplets were photographed under a phase contrast microscope. To quantify lipid accumulation, stained Oil Red O samples dissolved with 100% isopropanol and then the absorbance of solution was measured at 520 nm.

### 4.4. MTT Assay

3T3-L1 cells (2 × 10^4^ cells/well) were cultured in 24-well plates. After 24 h, cells were treated with various concentrations of RC-3095. After 24, 48 or 72 h, the MTT solution (0.5 mg/mL) was added to each well. After incubation for 3 h, the medium was removed and formazan dye was extracted by DMSO. The absorbance was detected at 495 nm.

### 4.5. Western Blot Analysis

Harvested cells were lysed in a RIPA buffer (50 mM Tris; pH 7.5, 150 mM NaCl, 0.5% sodium deoxycholate, 0.1% SDS, 2 mM EDTA, 1% Triton X-100) with a protease inhibitor cocktail (Sigma-Aldrich) and the protein concentration was measured by BCA assay. Equal amounts of protein (30 μg/lane) were separated using SDS-PAGE, and transferred to a nitrocellulose membrane (GE Healthcare, Marlborough, MA, USA). The membrane was blocked with 5% skim milk in TBS containing 0.1% Tween 20 for 1 h and probed with appropriate antibodies. The signal was detected using enhanced chemiluminescence (ECL) reagent (GE Healthcare Life Sciences).

### 4.6. Enzyme-Linked Immunosorbent Assay (ELISA)

The amounts of GRP protein in plasma and adipose tissue-conditioned medium (ATCM) were determined by ELISA according to the manufacturer’s instructions (Cusabio, Carlsbad, CA, USA). The absorbance of the samples at 450 nm was measured using an ELISA reader (Dynex), and the GRP amounts were determined by interpolating the values on to a standard curve generated as per the manufacturer’s instructions.

### 4.7. RT-PCR and Real-Time PCR Analysis

Total RNA was isolated with the Hybrid-R kit (Geneall, Seoul, Korea) according to the protocol recommended by the manufacturer. cDNA was synthesized with 2 μg of total RNA using the reverse transcription kit (Promega, Madison, WI, USA). For RT-PCR analysis, cDNA was amplified with following primers: GRP, 5′-TCTTGCTGTTGGCTCTGGTC-3′ and 5′-GGATCCCAAGTAGGCTGGAG-3′; GRP-R, 5′-TGGGAGACCTACTGCTGCTG-3′ and 5′-TTTAGGGTGCAGCTCATTGG-3′; GAPDH, 5′-CAACTCCCTCAAGATTGTCAGC-3′ and 5′-GGGAGTTGCTGTTGAAGTCACA-3′. Quantitative RT-PCR was performed with the AB7500 instruments (Applied Biosystems, Foster City, CA, USA) using SYBR green PCR master mix (Applied Biosystems). The primer sequences for real-time PCR were as follows: 36b4, 5′- TGGGCATCACCACGAAAATC-3′ and 5′-TTCAGCATGTTCAGCAGTGTGG-3′; C/EBP-β, 5′-GGGGTTGTTGATGTTTTTGG-3′ and 5′-CGAAACGGAAAAGGTTCTCA-3′; C/EBP-δ, 5′-GGAACACGGGAAAGCATGA-3′ and 5′-GGGTTAAGCCCGCAAACATTA-3′; PPAR-γ, 5′-TGGAATTAGATGACAGTGACTTGG-3′ and 5′-CTCTGTGACGATCTGCCTGAG-3′; GRP-R, 5′-TGATTCAGAGTGCCTACAATCTTC-3′ and 5′-CTTCCGGGATTCGATCTG-3′.

### 4.8. Chromatin Immunoprecipitation (ChIP) Assay

ChIP analysis was performed with the ChIP assay kit (Millipore, Burlington, MA, USA), according to the manufacturer’s protocol. Immunoprecipitation was performed with control IgG and anti-CREB (Abcam, Cambridge, UK) antibodies. The region containing CRE binding sites within the mouse *GRPR* promoter was amplified using PCR with specific primers. The oligonucleotide primers for PCR were as follows: CRE1 (−4737–−4729), 5′-TGAAAAGTCAAGAGTAGTGA-3′ and 5′-TGATGACAAGGTGATGTGCT-3′; CRE2 (−506–−499), 5′-TTGGAGAAAGCAGGCAGCTG-3′ and 5′-TTCCCTGATTTGTAGAACCT-3′. PCR was run for 35 cycles and amplicons were analyzed by 2% agarose gel electrophoresis. Independent experiments were repeated at least twice. Individual ChIP assays were repeated 3 times.

### 4.9. RNA Interference Experiment

The CREB siRNA and control siRNA were purchased from Santa Cruz Biotechnology and Bioneer (Daejeon, South Korea), respectively. For transfection with Amaxa Nucleofector (Lonza, Basel, Switzerland), cells were prepared at a density of 1 × 10^6^ cells in 100 μl of nucleofector solution. The solution was contained with 200 pM of CREB siRNA or control siRNA. The cells were transferred into the electroporation cuvette. After electroporation using Amaxa Nucleofector, the cells were cultured in 35-mm culture plates with fully supplemented medium.

### 4.10. High-Fat Diet (HFD)-Induced Obese Model and Experimental Protocols

Male Sprague-Dawley rats (SD, 6 weeks of age), weighing 210–230 g, were obtained from Samtako, Osan, Korea. After three days of acclimatization, the rats had free access to either a standard rodent chow (SD, fat content, 11% of energy), or a high-fat diet (fat content, 60% of energy), based on lard (HF-L), olive oil (HF-O), coconut fat (HF-C) or fish oil (derived from cod liver, HF-F). Weight gain and food intake were monitored once a week. After 14 weeks, the animals were euthanized after an overnight fast (16 h). Venous blood was drawn from the heart into EDTA-coated vials, and plasma was prepared and stored at −80 °C pending further ELISA analysis. Next, aortas were removed and then, perivascular adipose tissue (PVAT) was dissected along the whole aorta and weighed. Four-hundred milligrams of fat tissue from each animal was collected and conditioned at 37 °C in 1 mL of serum-free DMEM-Ham’s F-12 medium with 0.2% BSA for 24 h. The adipose tissue-conditioned medium (ATCM) was centrifuged, frozen, and kept at −80 °C until use. Subcutaneous adipose tissue (SCAT) and visceral adipose tissue (VCAT) samples were collected in liquid nitrogen for lipid and mRNA analysis as described.

### 4.11. Statistical Analysis

Data were the mean ± SD obtained for at least three independent experiments. Statistical comparisons between groups were performed by the one-way ANOVA followed by the Student’s *t* test.

## Supplementary Materials

The following are available online at http://www.mdpi.com/1422-0067/19/12/3971/s1.

## Abbreviations

GRP	Gastrin-releasing peptide
GRP-R	Gastrin-releasing peptide receptor
CREB	Cyclic AMP response element binding protein
C/EBPs	CCAAT/enhancer binding proteins
PPAR-γ	Peroxisome proliferator-activated receptor-γ
