# Supplementary material for: Involvement of Gastrin-Releasing Peptide Receptor in the Regulation of Adipocyte Differentiation in 3T3-L1 Cells
Source: Int J Mol Sci. 2018 Dec 10;19(12):3971. doi: 10.3390/ijms19123971 (PMC6321486; doi:10.3390/ijms19123971)
Supplement: Supplementary file 1 [file ijms-19-03971-s001.pdf]

▶ Supplemental Figure 1

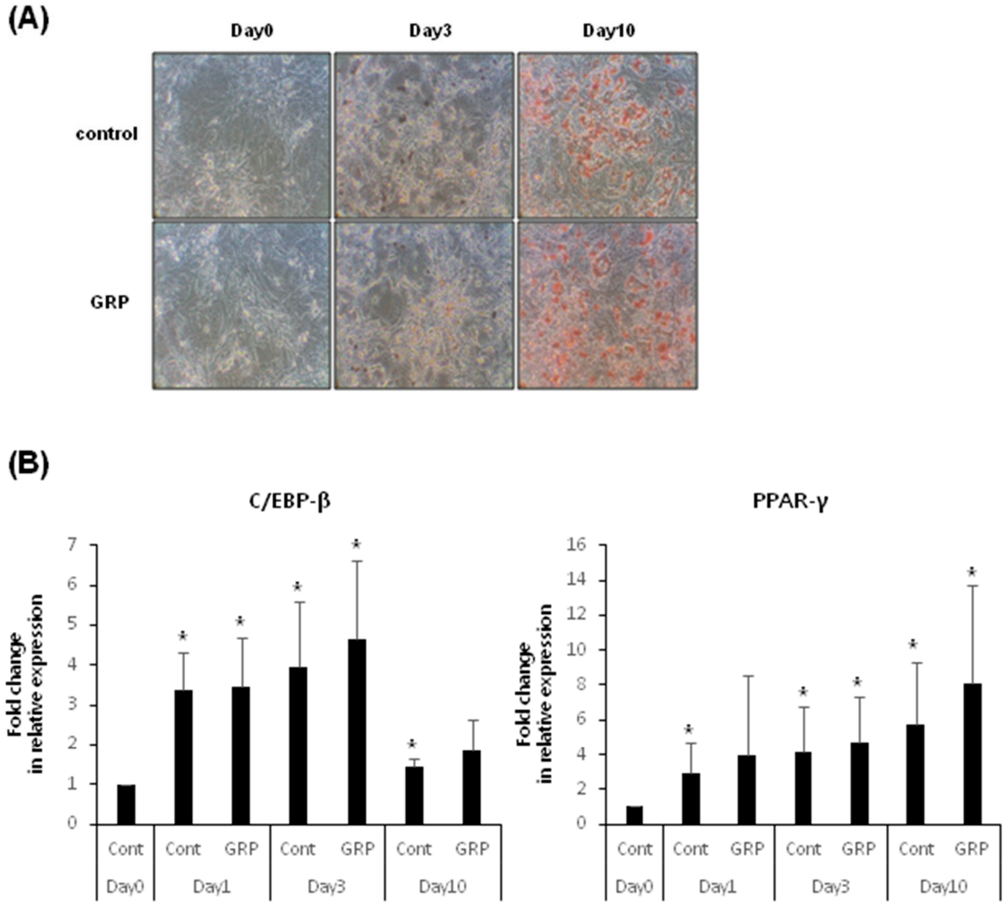

Supplemental figure 1. The effect of GRP on adipocyte differentiation of 3T3-L1 cells. (A) During differentiation, GRP (100 nM) was co-treated with DMI (DEX, IBMX, and insulin). Adipogenic cells were observed and stained with Oil Red O under a phase contrast microscope. (B) The effect of GRP on adipogenic differentiation markers was analyzed by Real-time PCR using primers specific to *C/EBP-β* or *PPAR-γ*. *$P < 0.05$ compared to Day 0.
